# Vagus Nerve Stimulation Paired with Tones for the Treatment of Tinnitus: A Prospective Randomized Double-blind Controlled Pilot Study in Humans

**DOI:** 10.1038/s41598-017-12178-w

**Published:** 2017-09-20

**Authors:** Richard Tyler, Anthony Cacace, Christina Stocking, Brent Tarver, Navzer Engineer, Jeffrey Martin, Aniruddha Deshpande, Nancy Stecker, Melissa Pereira, Michael Kilgard, Chester Burress, David Pierce, Robert Rennaker, Sven Vanneste

**Affiliations:** 1 University of Iowa Department of Otolaryngology-Head and Neck Surgery and Communication Sciences and Disorders, The University of Iowa, Iowa City, IA USA; 2 Department of Communication Sciences & Disorders, Wayne State University, Detroit, MI USA; 3 Department of Communicative Disorders and Sciences, University at Buffalo, Buffalo, NY USA; 4 MicroTransponder, Inc., 2802 Flintrock Trace, Suite 226, Austin, TX USA; 5 Callier Center for Communication Disorders, School for Behavioral and Brain Sciences, University of Texas at Dallas, Richardson, TX USA; 6 Department of Speech-Language-Hearing Sciences, Hofstra University, Hempstead, NY USA; 7 Texas Biomedical Device Center, University of Texas at Dallas, Richardson, TX USA; 8 Lab for Clinical and Integrative Neuroscience, School for Behavioral and Brain Sciences, University of Texas at Dallas, Richardson, TX USA

## Abstract

The aim of the pilot study was to evaluate the effect of Vagus Nerve Stimulation (VNS) paired with sounds in chronic tinnitus patients. All participants were implanted and randomized to a paired VNS (n = 16) or control (n = 14) group. After 6 weeks of home therapy, all participants received paired VNS. The device was used on 96% of days with good compliance. After 6 weeks, the paired VNS group improved on the Tinnitus Handicap Inventory (THI) (p = 0.0012) compared to controls (p = 0.1561). The between-group difference was 10.3% (p = 0.3393). Fifty percent of the participants in the paired VNS group showed clinically meaningful improvements compared to 28% in controls. At one year, 50% of participants had a clinically meaningful response. The therapy had greater benefits for participants with tonal and non-blast induced tinnitus at the end of 6 (24.3% vs. 2%, p = 0.05) and 12 weeks (34% vs. 2%, p = 0.004) compared to controls with 80% and 70% responding at 6 months and 1 year, respectively. Adverse effects were mild and well-tolerated and the therapy had a similar safety profile to VNS for epilepsy. VNS paired with tones may be effective for a subgroup of tinnitus patients and provides impetus for a larger pivotal study.

## Introduction

Tinnitus is the perception of a chronic ringing or buzzing sound in the absence of external stimulation. The reactions to tinnitus have been categorized into four primary functions of daily living activities: emotions, hearing, sleep and concentration^1–4^. Several counseling and sound-based therapies have been developed to help some patients manage these reactions^5–10^. Those that incorporate cognitive behavior therapy principles have received particular attention^11–13^. However, many patients with chronic tinnitus continue to remain refractory to treatments.

The psychological model of tinnitus proposed by Dauman and Tyler^14^ distinguishes the physiologic mechanisms of tinnitus from the reactions to tinnitus^15^. Preece *et al*.^16^ categorized mechanisms into three broad categories: (1) deafferentation and central- induced changes, (2) increases in spontaneous activity, and (3) increases in cross-fiber correlation^17^. One of the proposed mechanisms of tinnitus is generally thought to arise from hyperactive neurons in the central auditory system^18–22^. Neurons deprived of auditory input respond to the same frequencies as neighboring neurons that receive input from undamaged parts of the cochlea^22^. This results in an increase in the number of neurons in the primary auditory cortex that respond to a narrow range of frequencies. Subsequently, spontaneous activity and synchrony set in, which can create the perception of tinnitus^23,24^.

Pairing vagus nerve stimulation (VNS) with tones can reverse this maladaptive neuroplasticity in an animal model^25^. VNS promotes neuroplasticity that is specific to the paired experience by triggering a timed burst of neurotransmitters^25^. The tones paired with VNS were specifically chosen to asynchronously activate auditory neurons surrounding the tinnitus frequency (excluding the tinnitus frequency), thereby reducing the synchronous activity of neurons in the hyperactive tinnitus region^26,27^.

The first feasibility study using this conceptual framework was an open-label, 10-patient study^28^. Stimulation settings were identical to those used in the animal experiments^25^. After 4 weeks of daily VNS-tone pairing, tinnitus symptoms improved in 40% of the participants and this improvement lasted for at least 2 months^28^.

The aim of the pilot trial was to further explore the safety and efficacy of paired VNS therapy in a double-blind, randomized, controlled study in a larger sample of participants (n = 30), for a longer period of time, and at four independent sites. This pilot study was not powered a priori to detect efficacy. The main intent was to provide both safety and efficacy information to support the design and implementation of a pivotal study for US market approval.

## Results

Sixty-two individuals were screened and thirty were implanted (see Methods). All participants followed the standard protocol visit schedule. No participants discontinued therapy during the randomized portion of the study and all participants elected to continue treatment during the long-term portion of the study. There were no significant protocol deviations that impacted the study results or conclusions. Baseline demographics of enrolled and implanted participants are provided in Table 1.

**Table 1:** Baseline demographics (mean and standard deviation) for paired VNS and control group for participants enrolled in the study.

Baseline characteristic	Paired VNS (n = 16)	Control (n = 14)	Statistics
Age (yrs.)	55.9 (7.6)	54.9 (9.1)	*t* = 0.33, *p* = 0.74
Gender (frequency per category)	Male: 15/Female: 1	Male: 10/Female: 4	χ^2^ = 2.68, *p* = 0.10
Tinnitus duration (yrs.)	18.8 (17.1)	10.1(10.3)	*t* = 1.65, *p* = 0.11
Tinnitus pitch (Hz)	6460 (2283)	7853 (2931)	*t* = 1.39, *p* = 0.18
Number of previous therapies tried	3.9 (2.2)	3.8 (1.9)	*t* = 0.20, *p* = 0.84
Tinnitus Handicap Inventory (THI)	52.5 (22.6)	53.6 (18.5)	*t* = 0.15, *p* = 0.88
Tinnitus Handicap Questionnaire (THQ)	58.8 (18.3)	57.3 (19.3)	*t* = 0.21, *p* = 0.83
Tinnitus Functional Index (TFI)	60.9 (17.5)	61.5 (17.1)	*t* = 0.10, *p* = 0.92
Loudness Severity (0–100)	69.5 (19.8)	73.5 (21.3)	*t* = 0.54, *p* = 0.59
Minimal Masking Level (dB)	48.10 (18.13)	61.73 (22.96)	*t* = 1.82, *p* = 0.08
Loudness Match (dB)	53.38 (20.32)	59.34 (17.77)	*t* = 0.85, *p* = 0.40
BDI (Beck Depression Inventory)	8.14 (6.83)	10.38 (7.77)	*t* = 0.83, *p* = 0.41

### Safety

In general, adverse events were mild and well-tolerated. No new or unexpected events were observed. Moderate adverse events involved two participants who experienced iatrogenic vocal-cord paralyses (hoarseness or voice weakness) after implant that lasted longer than 12 weeks. In one of those participants, the affected side returned back to normal while in the other participant, hoarseness and vocal cord movement improved but the vocal cord paralyses did not recover completely. This participant was offered the possibility of gel injections and speech therapy but declined both. One of these events occurred with a surgeon new to VNS surgery. One lead fracture occurred which required replacement surgery after re-engineering of the lead. There were no other related serious adverse events.

### Compliance

All 30 implanted participants completed the randomized portion of the study (6 weeks) and all participants continued performing the therapy daily for the next 6 weeks (12 weeks total). Participants continued to do the therapy periodically afterwards and were evaluated at quarterly time-points for up to a year. For the first 6 weeks, the device was used on 96% of total possible days. Participants received an average of 273 (±38 SD) stimulations per day (approximately 2.5 (±0.1 SD) hrs/day) during the first 6 weeks.

The highest number of missed days for a single participant was 31% (13 of 42). The protocol required the device to be used for at least four (4) out of every seven (7) days (57%; or 18 or fewer missed days) with no more than three (3) unused days in a row; all participants met the compliance criteria. There were no compliance issues with the system, even though use required that the participant actively start treatment (by starting their computer and hitting the start button) and be near their laptop for 2.5 hours. The stimulation parameters were confirmed upon return clinic visits by establishing communication with the IPG.

Twenty-seven (27) of thirty (30) participants (90%) kept their device and equipment through at least 6-months. Two participants had their device explanted and one participant returned his equipment prior to 24 weeks. The two participants that were explanted did have an improvement on their tinnitus symptoms (THI decrease of 39.13% and 55.17% respectively after 12 weeks of treatment). The participant that returned the device prior to 24 weeks had only a 2.7% decrease on the THI after 12 weeks. An additional participant had their device removed after 10 months. Twenty of 30 (67%) participants routinely used the device through 12 months. An additional 5 participants still had the device implanted for sporadic use (83.3% used the device at least some during the long-term study). Only 5 participants (3 participants were explanted and 2 participants returned the equipment) stopped using the system within 1 year (17%).

### Efficacy

**Figure 1:**
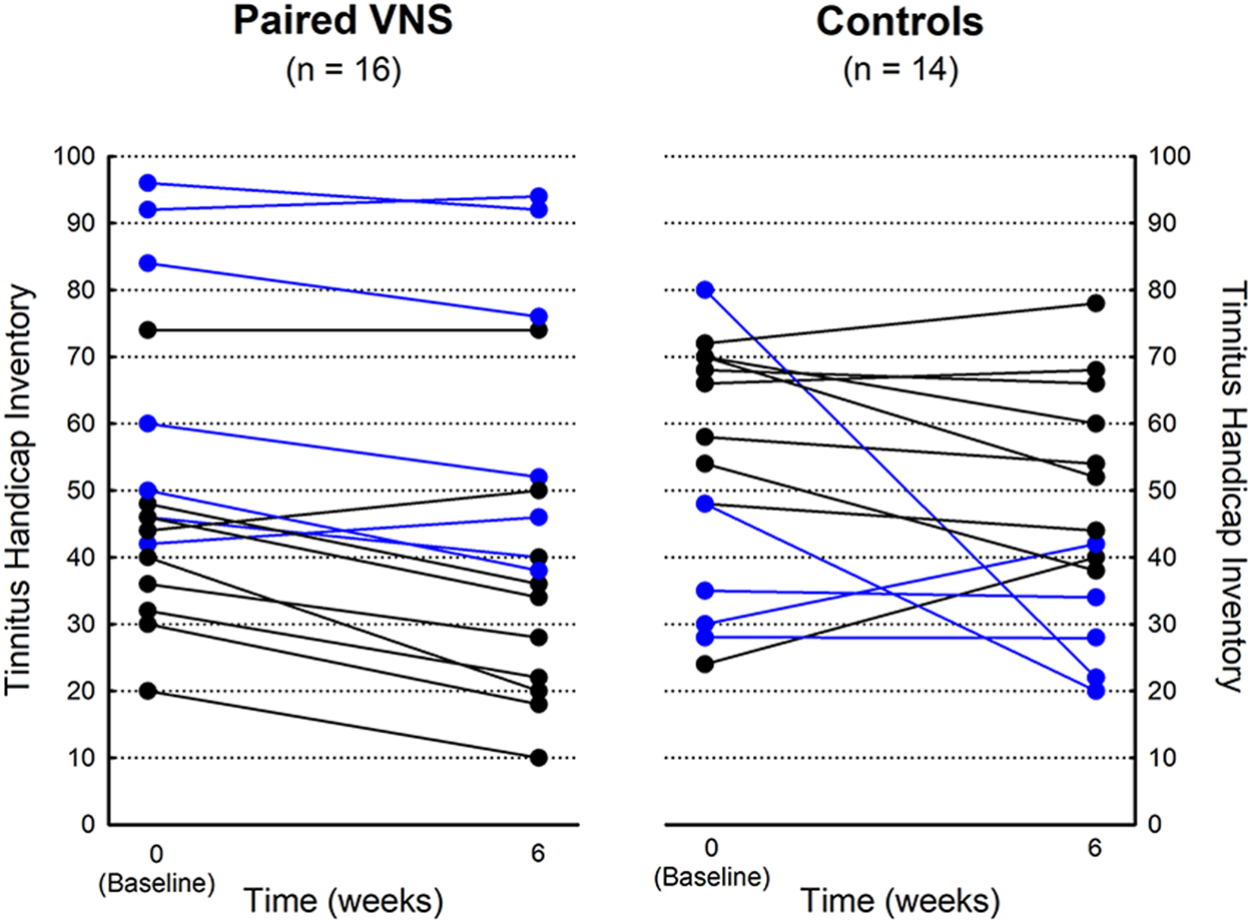
Individual changes in the Tinnitus Handicap Inventory (THI) score at baseline and 6 weeks for the paired VNS group (left; n = 16) and control (right; n = 14) group. Blue lines indicate participants with blast-induced and/or tonal tinnitus (n = 6 VNS; n = 5 controls).

The paired VNS group improved on the THI (−17.7%; 95% confidence interval, −28% to −7.3%, *p* = 0.0012) at the end of the randomized portion of the study (6 weeks) while the control group did not (−7.3%; 95% confidence interval, −27.5% to 12.7%, *p* = 0.1561). The between-group difference was 10.3% (95% confidence interval, −10.49% to 31.12%, *p* = 0.3393) (Table 2). The median (interquartile range) was −17.78% (−28.67, −2.08) for the paired VNS and −4.91% (−25.71, 3.03) for the control group at the end of 6 weeks. Figure 1 shows the individual participant data for the paired VNS and control group. There was wide variability in the THI response with some participants showing a decrease while others showed no change. A wide range of tinnitus severity was also observed at the baseline visit. THI scores ranged from 20 to 96 in the paired VNS group and 24 to 80 in the control group. Note that one participant in the control group displayed a very large decrease between baseline and 6 weeks and then an increase in the THI score between 6 and 12 weeks. The reason for this variability is unclear. The participant had a high THI score at baseline (80), indicating someone who was greatly distressed or anxious.

**Table 2:** Mean and Confidence interval data for outcome measures at the end of the randomized portion of the study (6 weeks).

Outcome Measure	Paired VNS change from baseline to 6 weeks (n = 16) (95% CI)	Control change from baseline to 6 weeks (n = 14) (95% CI)	Difference between groups (lower CI, upper CI)
THI (%)	−17.7 (−28 to −7.3)^*^	−7.3 (−27.5 to 12.7)	10.32 (−10.49 to 31.12)
THQ	−2.5 (−8.3 to 3.3)	−7.5 (−15.8 to 0.7)	−5.03 (−14.57 to 4.51)
TFI	−2.03 (−7.1 to 3.1)	−7.5 (−15.5 to 0.7)	−5.35 (−14.29 to 3.60)
Loudness severity	−6.69 (−13.26 to −0.11)	−8.5 (−22.6 to 5.5)	−1.88 (−16.09 to 12.32)
Loudness match	1.06 (−2.3 to 4.4)	0.36 (−7.9 to 8.6)	−0.71 (−8.85 to 7.44)
MML	3.5 (−0.4 to 7.5)	−3.8 (−11.9 to 4.1)	−7.46 (−15.70 to 0.77)

*p < 0.05.

Responder rate for the THI was defined as percentage of participants who showed a clinically meaningful response on the THI^29^. At the end of 6 weeks, 50% (8/16) of the participants in the paired VNS group showed a >20% improvement on the THI compared to 28% (4/14) in controls (20% cut-off: χ^2^ = 1.43, *p = *0.23).

After the 6 weeks of the blinded portion of the study, both groups were unblinded. After this time point, participants in the paired VNS group continued to receive the paired VNS while the control (unpaired) group crossed-over to receive paired VNS. Once unblinded, clinicians had the option to use different stimulation parameters (e.g., increasing or decreasing the stimulus intensity, changing the stimulation pulse width or interval between paired stimulations).

A repeated measures ANOVA was performed with condition (paired VNS vs. control) as the between-subjects’ variable and the THI at baseline, 6 weeks, and 12 weeks as the within-subjects’ variable. This analysis revealed a main effect for THI (*F* = 6.18, *p* = 0.006, *η* ^2^ = 0.31) demonstrating that in comparison to baseline (*M* = 53.03, *SD* = 20.45), a decrease was obtained after 6 weeks (M = 45.87, SD = 22.13, p = 0.023) and 12 weeks (M = 43.73, SD = 23.68, p = 0.004). We did not observe the main effect for condition (paired VNS vs. control) (*F* = 0.001, *p* = 0.97, *η* ^2^ < 0.001) or an interaction effect (*F* = 0.10, *p* = 0.91, *η* ^2^ = 0.007). The paired VNS group had a mean suppression of 17.71% (*Md* = 17.78%, *SD* = 19.45) on the THI, while the control group had a mean suppression of 7.39% (*Md* = 4.92%, *SD* = 34.97) after 6 weeks of treatment (*t* = 1.02, *p* = 0.32). Analysis for THQ, TFI, loudness severity, loudness match and MML are shown in Table 3.

**Table 3:** A repeated measures analysis for outcome measures THI, THQ, TFI, Loudness severity, MML and Loudness Match (LM).

		*F*	*p*	*η* ^2^	Baseline	Week 6	Week 12
THI	Main effect: time	5.98	0.007	0.31	49.93^a^	45.87^a,b^	43.73^b^
	Main effect: condition	0.003	0.96	0.001	—	—	—
	Interaction effect	0.10	0.94	0.007	—	—	—
THQ	Main effect: time	3.81	0.035	0.22	58.11^a^ (18.47)	53.25^b^ (20.57)	50.73^b^ (20.20)
	Main effect: condition	0.33	0.57	0.01	—	—	—
	Interaction effect	0.83	0.45	0.06	—	—	—
TFI	Main effect: time	4.59	0.019	0.25	61.19^a^ (17.02)	56.67^b^ (20.64)	53.44^b^ (18.87)
	Main effect: condition	0.30	0.59	0.01	—	—	—
	Interaction effect	1.01	0.38	0.05	—	—	—
Loudness Severity	Main effect: time	2.55	0.10	0.16	—	—	—
	Main effect: condition	0.15	0.70	0.005	—	—	—
	Interaction effect	0.06	0.95	0.004	—	—	—
MML	Main effect: time	0.08	0.93	0.006	—	—	—
	Main effect: condition	1.77	0.19	0.06	—	—	—
	Interaction effect	1.96	0.16	0.13	—	—	—
Loudness Match	Main effect: time	0.014	0.97	0.001	—	——	—
	Main effect: condition	0.14	0.71	0.005	—	—	—
	Interaction effect	1.00	0.38	0.07	—	—	—

^a,b^ *p* < 0.05, Bonferroni corrected.

### Long-Term Analysis

Fifty percent (8/16) of the participants in the paired VNS group exhibited a clinically meaningful improvement (>20% improvement) on the THI at the end of 6 weeks and 56% responded after 12 weeks. In the control group, only 28% (4/14) of the participants responded at the end of 6 weeks. After these participants crossed over to receive paired VNS, the responder rate increased to 43% at 12 weeks. Participants were followed up to one year and outcome measures and responder rates from both groups were pooled at 6 months and 1 year since both groups were receiving paired VNS therapy for the long-term portion of the study (Supplementary Tables 4 and 5).

At 6 months, responder rates for participants in both groups that continued to receive paired VNS were 56% (n = 16), 35% (n = 17) and 52% (n = 17) for the THI, THQ and TFI respectively. At 1 year, responder rates were 50% (n = 16), 64% (n = 17) and 50% (n = 16) for the THI, THQ and TFI, respectively. It should be noted that of the 14 participants in the VNS group and control group that responded to the paired VNS therapy during their first 6 weeks of paired VNS (8/16 VNS, 6/14 cross-over controls), eight participants used the device until one year and 6/8 (75%) continued to be responders at one year (average decrease of 54% on the THI).

An earlier study suggested using a 7-point absolute cut-off for the THI as being clinically meaningful^30^. Using this cut-off, responder rates for the THI were 62% at 6 months and 50% at 1 year. Some authors have suggested using a 13-point cut-off for the TFI^31^. Using this cut-off, responder rates were 35% and 37% at 6 months and 1 year, respectively. A summary of all responder rates is provided in the Supplementary Materials. The changes on outcomes at 1 year from available participants are shown in Table 4.

**Table 4:** Long term change in scores for outcome measures one-year post-therapy (both groups received Paired VNS therapy after the initial 6 weeks of the randomized portion of the study).

	Change from baseline at one year Mean (lower CI, upper CI) (n, participants)	One sample Test P value
Tinnitus Handicap Inventory	−19.39 (−37.99, −0.79), (16)	0.0421*
Tinnitus Handicap Questionnaire	−11.99 (−19.72, −4.26), (16)	0.0048*
Tinnitus Functional Index	−9.98 (−19.74, −0.21), (16)	0.0459*
Loudness Severity	−19.41 (−34.01, −4.82), (17)	0.0123*
Loudness Match	−5.33 (−11.62, 0.96), (18)	0.0915
Minimum Masking Level	−3.34 (−13.13, 6.46), (17)	0.4807

*p < 0.05.

**Figure 2:**
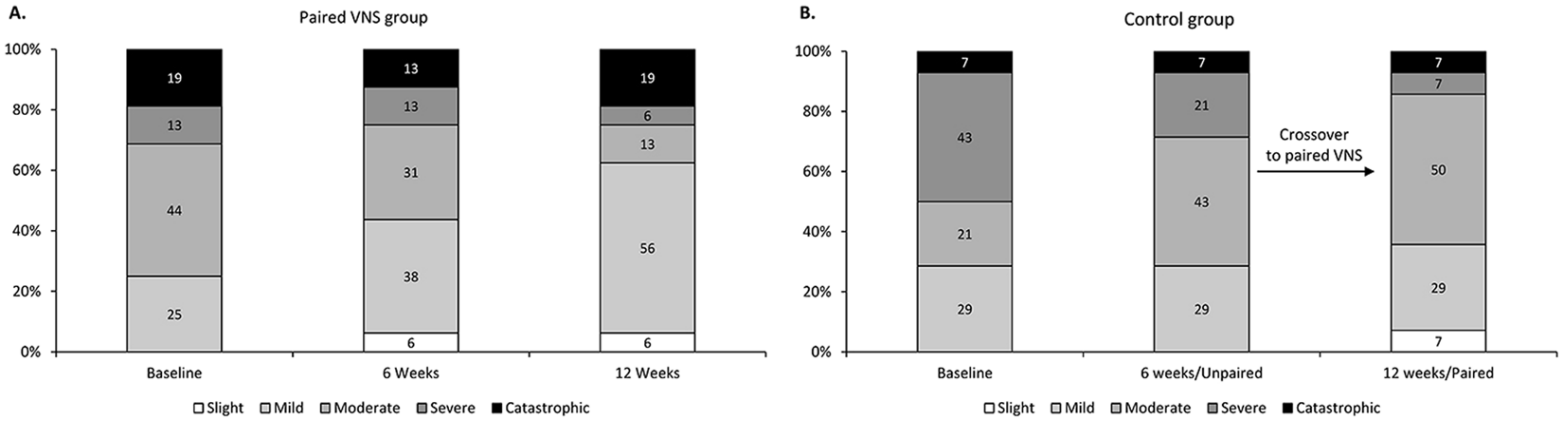
The THI changes at baseline, 6 and 12 weeks of treatment based on the severity of tinnitus. The severity or THI grade is generally categorized as Slight (0–16), Mild (18–36), Moderate (38–56), Severe (58–76) and Catastrophic (78–100).

Next, we looked at the proportion of participants that dropped a category on the THI scale as they progressed through therapy. For the paired VNS group (n = 16), 43.75% of participants had moderate tinnitus at baseline, which decreased to 12.5% at 12 weeks. This was accompanied by large increase in the proportion of participants with mild tinnitus from 25% at baseline to 56% at 12 weeks. In the unpaired group (n = 14), a large proportion of participants had severe tinnitus prior to treatment (42.86%). This dropped to 7.14% at the end of 12 weeks (this period included the paired VNS therapy) (Fig. 2).

### Subgroup Analysis

We evaluated the responses within this tinnitus population to determine if certain participants responded better to the therapy than others. VNS treatment appeared to have greater benefits for participants that did not have hissing tinnitus and/or blast-induced tinnitus. After excluding this subset of participants (n = 11 participants excluded), a 24.3% improvement was obtained on the THI in the paired VNS group, compared to only 2% in the control group at the end of 6 weeks (*p* = 0.05). After 12 weeks, the THI improved in the paired VNS group by 34% compared to 2% in controls (*p* = 0.004). Responder rate was 80% and 70% at 6 months and one year respectively. The average THI decrease for these responders was 44% and 40% respectively, in this subpopulation.

## Discussion

This pilot study was significantly different from the earlier open label feasibility study in several ways. First, this was a double-blind, randomized controlled trial. Second, participants *in both groups* were implanted and controls crossed over (after 6 weeks) to receive the paired VNS therapy. Third, participants continued to use the device over one year and were followed up with quarterly assessments. Finally, the earlier open label feasibility study used an implanted lead with an *external* stimulator. Participants had to be in the clinic each day to receive stimulation for 4 weeks (20 weekdays). The current study had the stimulator and lead both implanted such that therapy could be delivered at home.

There was a wide variability in the responses across participants. For example, two participants showed large decreases in the THI during the control portion of the study. It is possible that the improvement may have resulted from a placebo effect or that VNS may be modulating the emotional component of the tinnitus^32–38^. When considering the latter, it should be noted that the use of VNS in this study is distinctly different from the use of VNS for epilepsy and patients with major depression, where VNS is given 24 hours per day, seven days a week, and the stimulation is not paired with any specific sensory input. In contrast, in this study, substantially less VNS was administered compared to epilepsy^32^ and depression^33^ studies (2.5 minutes of stimulation/day for tinnitus vs. 144 minutes of stimulation/day for epilepsy/depression). Therefore, it is unclear whether the unpaired VNS in the control group may have contributed to the therapeutic benefits in some participants and/or was a placebo effect. Our preclinical studies had previously demonstrated that unpaired VNS did not reverse the tinnitus percept or plasticity in a rat animal model of tinnitus^25^. Moreover, no differences in depression scores (BDI) were obtained between the groups and no overall decrease in BDI was observed in the long term. Subsequent studies will explore the timing of paired VNS to have a better understanding of the mechanism of action.

Our results show that VNS was associated with reductions in tinnitus severity in a subgroup of tinnitus patients (tonal and non-blast induced tinnitus). In animal studies, tinnitus was evaluated in a gap-detection behavior model whereby rats underwent noise exposure and were subsequently tested for tonal tinnitus percept^25^. Therefore, the study model was a noise exposure model (non-blast induced) with tonal tinnitus. It is possible that the pathophysiology of blast-induced tinnitus is different from tinnitus due to prolonged noise exposure. It is also possible that a different pairing paradigm may be needed for hissing quality of tinnitus. Given the wide heterogeneity of tinnitus pathophysiology and symptoms, paired VNS therapy may be effective within certain subgroups.

We did not observe large consistent decreases for the MML or loudness match measures. Use of psychoacoustic measures such as the MML have been problematic in earlier studies^39,40^ and loudness matching is known to be subject to learning effects^41^. In this study, there was high variability in MML measurements across baselines with an average difference of 7.4 dB (range 6.2 to 29 dB), making interpretation of the results difficult.

VNS is known to be a safe and well-tolerated procedure that has been performed in approximately 100,000 patients worldwide for the treatment of severe depression and epilepsy^42,43^. Two participants reported hoarseness or vocal weakness lasting more than 12-weeks. As in our case, this has previously been associated with VNS surgery and is comparable to the rates seen in other early VNS studies for the treatment of epilepsy. Nevertheless, the events did not interfere with compliance. Future studies will assess vocal cord function prior to surgery and use experienced VNS surgeons with enhanced surgical training.

All participants completed the randomized phase of the study and side effects due to stimulation were minimal (i.e. hoarseness). The pairing of VNS with tones revealed no new types of adverse effects, suggesting that the safety profile is similar to VNS for epilepsy and depression.

Although the therapy was time consuming (2.5 hours a day, 7 days a week), compliance during the first 6 weeks of the study was very good. After the first 12 weeks, participants could use the device for differing amounts, although typically for 1.5 to 2.5 hours a day, for 3–7 days a week. Only 2 out of 30 participants were explanted after 6 months. Both participants had a decrease on their THI between 30–60% at 6 months suggesting that both participants believed the benefit had plateaued. One participant returned the computer after 6 months, due to a weak improvement on the THI. Most other participants elected to remain implanted and try new or different settings. It is possible that periodic use of the device may have accounted for minimal or late onset of improvement in some participants.

In this study, the stimulation settings were identical to those used in the animal and the feasibility studies^25,28^. However, it is not known whether the stimulation parameters used to activate the human vagus nerve are optimal. Recent animal research demonstrated that moderate intensity of VNS (0.4 and 0.8 mA) drive greater plasticity compared to VNS at higher intensities (1.2 and 1.6 mA)^44^. However, it is possible that different frequencies, different pulse widths or amplitudes might reveal better results^45^.

Individuals with tinnitus may certainly benefit from counseling and sound therapy. However, it should be noted that the individuals enrolled in this study had tinnitus for 18 years on average and had tried several other therapies, making them a refractory population. The data here showed an improvement on all three questionnaires as well as loudness severity rating after one year of treatment. For responders, the average decrease in THI was 47% (in terms of absolute decrease, these responders had an average THI decrease of 20 points from baseline) at one year. Although not all individuals participated in the long-term follow-up, these results are encouraging and suggest a long-lasting therapeutic benefit.

A limitation of the study was the small number of participants and sites due to the pilot nature of the study. Potential clinical effects of promising new tinnitus interventions should be first tested in smaller trials, which can give important information on the effect size of the intervention and may help to identify subgroups of patients being more likely to respond to the tested intervention^46^. This information is necessary to design large prospective placebo-controlled clinical trials, which are costly and time consuming^46^. Based on the results from this study, a 11% difference between paired VNS and control group with a common standard deviation of 20 would indicate 53 participants per group (106 total participants) and would have 80% power. This pilot study serves as primary support for the design and implementation of a larger pivotal study (120 participants) for US market approval.

Given the heterogeneity of chronic tinnitus, it is unlikely that any single treatment will be effective for all patients with tinnitus^47^. The observation of a robust response in a subgroup of patients that corresponded most closely to our preclinical data suggests that paired VNS is associated with reductions in tinnitus severity for certain types of tinnitus. The results of this study demonstrate that VNS may be promising for a subgroup of patients with chronic tinnitus. The 120-subject pivotal study has been approved by the FDA to start enrollment.

## Methods

### Study Design

**Figure 3:**
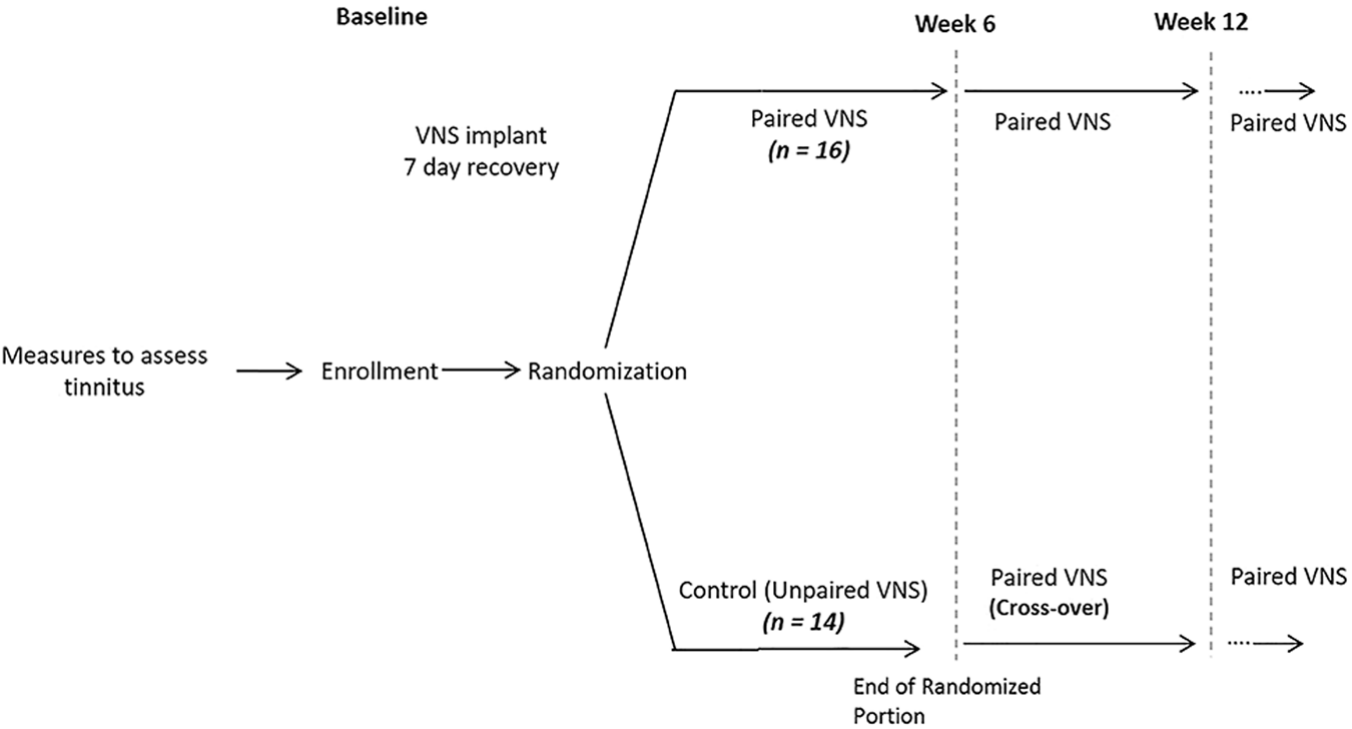
Study design. Participants were implanted with the device and randomized to either a paired VNS (n = 16) or control (n = 14) group. Participants performed the treatment at home for approximately 2.5 hours/day, 7 days/week. During the first 6-weeks, the implanted control group also received VNS but was unpaired from tones. After 6 weeks of blinded, controlled treatment, participants in the control group crossed over to receive paired VNS. After 6 weeks, participants in both groups received the paired VNS stimulation and were assessed until one year.

The study was a two-arm, double-blind, randomized, and controlled study in 30 participants with chronic sensorineural tinnitus. Participants in both groups were implanted with the VNS device and randomized to either a paired VNS (n = 16) or control (n = 14) group (Fig. 3). Participants performed the treatment at home for approximately 2.5 hours/day, 7 days/week, for 6 weeks. During the first 6 weeks, the VNS group received VNS paired with tones (discussed below). The control group also received VNS but was unpaired from tones. After 6 weeks, participants in the paired VNS group continued to receive paired VNS while participants in the control group crossed over to receive paired VNS. Both groups were followed up to a year.

Participants were enrolled at 4 centers in the United States (Supplementary Table 1). The trial was performed under an approved FDA Investigational Device Exemption (IDE, #G130140) and registered on clinicaltrials.gov (NCT01962558) on 9^th^ October 2013. Informed consent was obtained in compliance with the requirements set forth in U.S. Food and Drug Administration, Code of Federal Regulations Title 21.

With respect to safety, a sample of 30 participants allowed adequate power to detect the incidence of rare safety and device events. A sample of 30 participants yielded 95% probability that the study will reveal at least one occurrence of all events or complications that occur in participants at a rate of 9.5% or greater. In addition, implantation and follow-up of 30 participants for 6 weeks will yield 1,260 days of exposure. In this case, the threshold for detection decreases to a very unlikely event, one that occurs in only 0.2375% of days. In other words, if an event has a probability of 0.002375 or 1 event in 422 days of exposure, we have a 95% probability of observing this event in a sample of 30 participants with 1,260 days of exposure. As participants continue into the long-term portion of the study, the ability to detect rare events increases as the exposure increases.

### Participants and Enrollment

Sixty-two participants were screened across the study sites. Of the 62 participants, 32 did not proceed due to (1) participants decision, typically regarding the participants or a family member’s concern about surgery or time commitment for the study (15), discontinuation by the site after the study implant limit was reached (5), failure to meet ongoing tinnitus levels (4), medication contraindication (3), other medical issues (3) and excessive hearing loss (2). Thirty participants were enrolled and implanted which included 25 males and 5 females.

### Inclusion and Exclusion Criteria

Key inclusion criteria included individuals with sensorineural tinnitus who (a) were 22 to 65 years-of-age (b) had primarily a tonal quality to their tinnitus (c) had either unilateral or bilateral tinnitus (d) had experienced tinnitus for at least one year in duration (e) had engaged in at least one tinnitus therapy program and found it unhelpful. Key exclusion criteria included (a) acute or intermittent tinnitus (b) Meniere’s disease, retro-cochlear disease or evidence of active middle-ear disease (c) any active implanted device such as a pacemaker or other neurostimulator or any other investigational device or drug (d) Beck Depression Inventory (BDI) of 30 or greater (e) Any drug known to mimic, increase, or decrease release or removal of a diffuse neuromodulator, such as norepinephrine, dopamine, serotonin, benzodiazepines, acetylcholine, psychoactive medications or medication known to cause or increase tinnitus.

### Randomization and blinding

The contract research organization (CRO) performing the data analysis supplied the randomization for each participant to the site via a phone call with unblinded site personnel who performed the device programming and set the device to the proper group device settings. The participant initiated each therapy session but was unaware of the group allocation (there was a “start” button, but no information on the specific settings was shown to the participant). This allowed the blinded condition to be maintained, since participants were often unable to perceive VNS or only sporadically perceive VNS either via neck tingling or voice hoarseness. The audiologist performing the assessments on a specific participant was not allowed to operate the device settings for that participant so as to remain blinded to that participant’s settings. Participants were randomized using SAS (PROC PLAN) and stratified by site. Each site had its own randomization sequence, with an initial block of two followed by blocks of four. For each participant, the site called the CRO to receive the treatment assignment, taken sequentially from the randomization list for that site.

### VNS Implantation and Stimulation Parameters

Device implantation was typically performed under general anesthesia by an otolaryngologist. One participant, however, was implanted using local anesthesia, at their request. Details of the device implantation have been described previously^28^. After surgical implantation and approximately one week of recovery, participants were randomized to either the paired VNS group (n = 16) or to the active control therapy group (n = 14).

Participants were admitted to the surgical center/hospital on the morning of the device implantation and were discharged within 24 hours. The implantation involved placement of the lead’s stimulation electrodes on the left vagus nerve in the carotid sheath. The lead connector was then tunneled subcutaneously to a pocket created in the left ancillary or pectoral region where it was attached to the implantable pulse generator.

**Figure 4:**
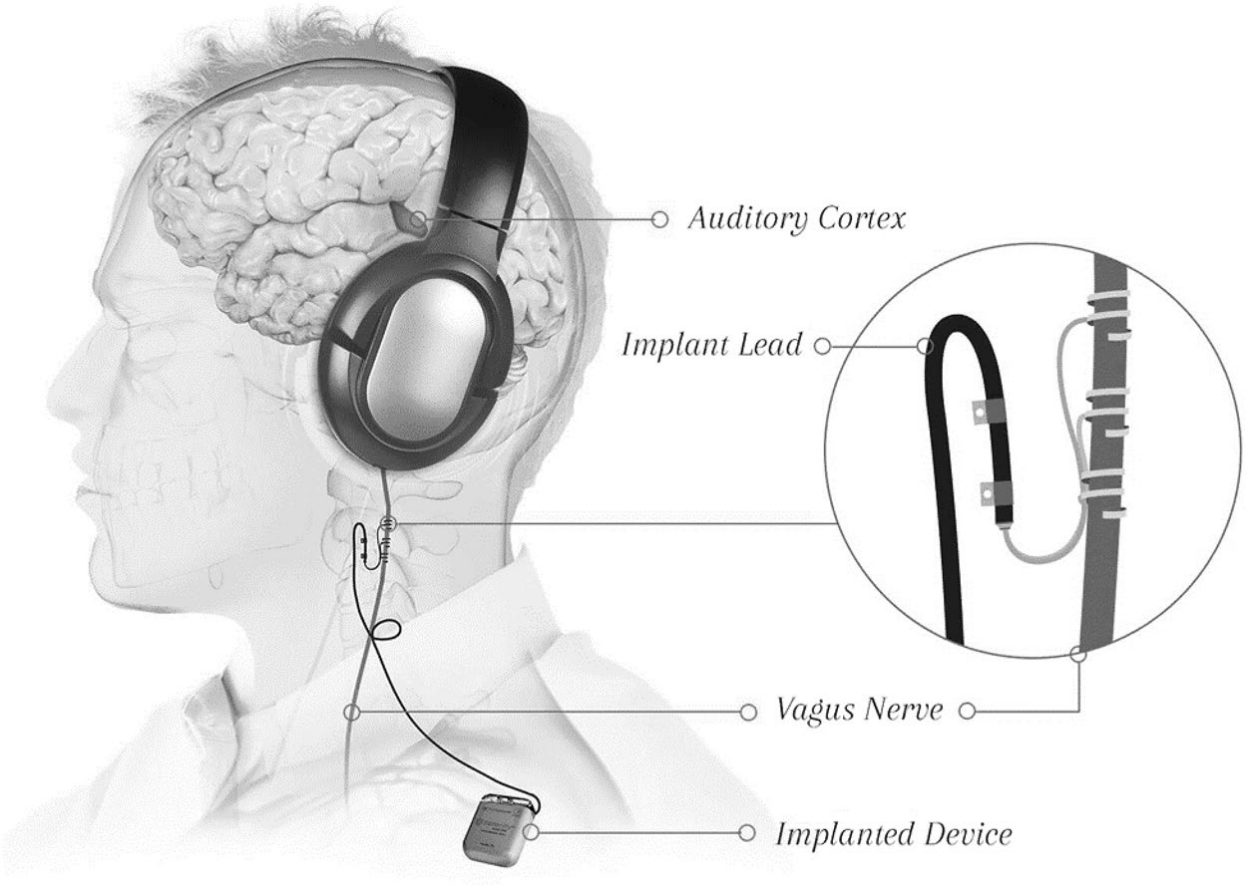
Setup using the Serenity® System that pairs Vagus Nerve Stimulation (VNS) with tones. The inset shows the electrode lead wrapped around the cervical vagus nerve. The device is the pulse generator that is implanted under the chest wall. The implant lead connects to the pulse generator. Image courtesy of *MicroTransponder*, *Inc*.

The device consisted of an implantable pulse generator (Model 1000 Serenity®), an implantable lead and electrode (Model 3000), and an external controller system (Fig. 4). The external controller system included a laptop computer (Dell Inspiron) with high quality circumaural headphones (Sennheiser, HD280-PRO), running the Tinnitus Application Programming Software (TAPS Model 4000) and an external controller. The external controller (Model 2000, connected to the laptop via USB) communicated wirelessly with the IPG stimulator. The software enabled the audiologist to program the stimulation parameters (amplitude (mA), frequency (Hz), pulse width (µs), duration (ms)), review captured participants’ programming history, and check lead impedance and battery status. The software also captured participants’ programming history.

**Figure 5:**
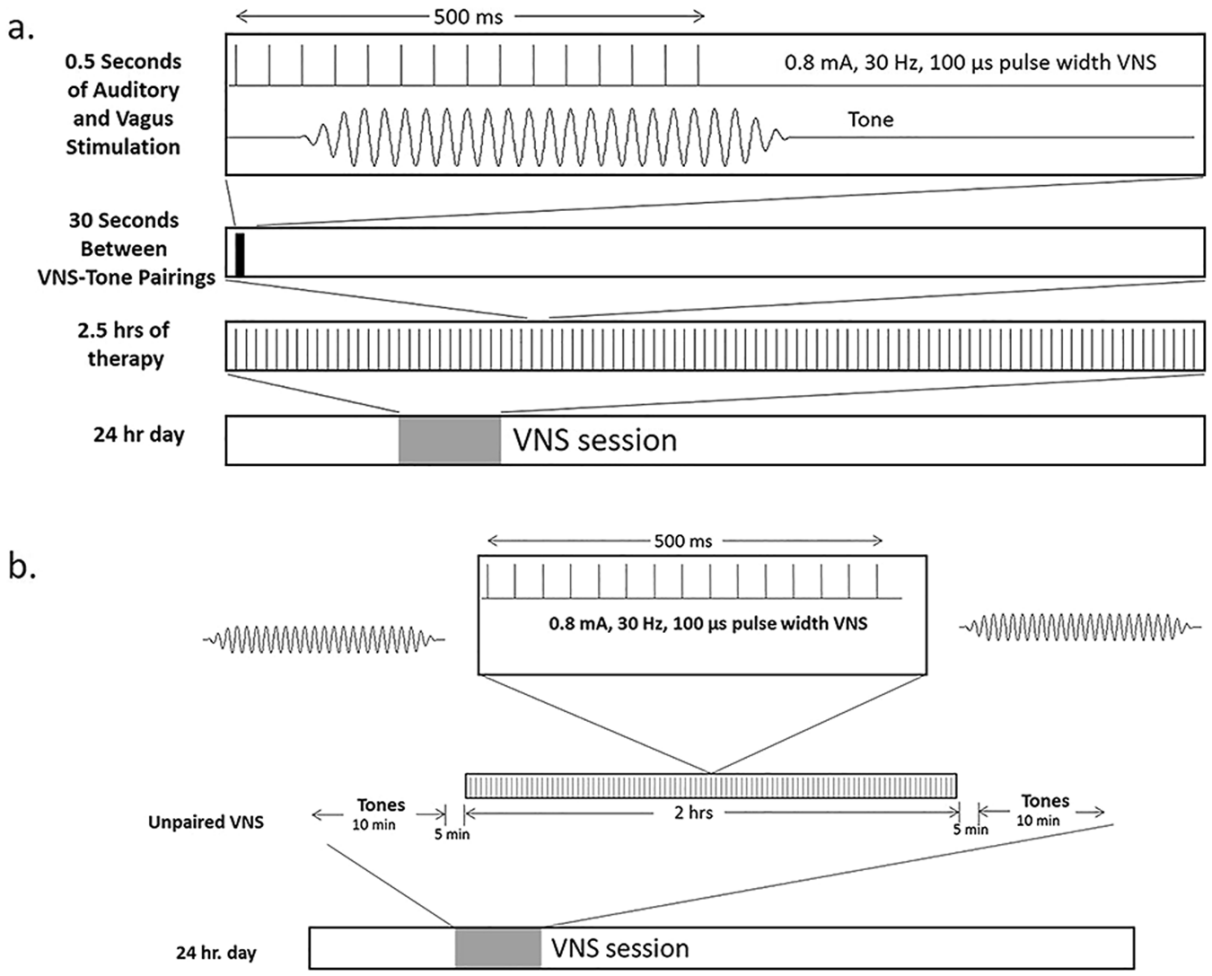
(**a**) Stimulation settings for paired VNS therapy. The lower panel shows the stimulation settings (0.8 mA, 30 Hz), which overlaps with the tone. Each VNS tone pairing was presented every 30 seconds, for approximately 2.5 hrs per session over a period of 24 hrs. (**b**) Stimulation settings for the Unpaired (Control) group. During each session, participants received 10 minutes of tones only, 5 minutes of silence and no VNS; 2 hours of VNS only; 5 minutes of silence and no VNS, and 10 minutes of tones only.

Participants started the therapy after approximately one week of recovery from surgery. Stimulation was delivered to the left vagus nerve since this is the most common practice in VNS for epilepsy and depression. However, since the upstream targets are bilateral, stimulation likely affects both sides of the cerebral hemispheres. Each VNS stimulation consisted of fifteen 0.8 mA, constant current, charge balanced pulses (100 µs pulse width, at 30 Hz). The duration of the VNS pulse train was 0.5 seconds (Fig. 5a). Each pulse train was delivered approximately every 30 seconds for 2.5 hours. In no instance were settings outside those used for VNS in epilepsy or depression (output currents were ≤3.5 mA, frequencies were ≤30 Hz, pulse widths were ≤1000 µs and duty cycles (ON time / OFF times) ≤50%.

In the paired VNS group, each 0.5 s VNS pulse was presented simultaneously with a 0.5 s tone every 30 s for 2.5 hrs. Therapy tones excluded one or more of the participant’s tinnitus frequencies. The tones paired with VNS were at least ½ octave away from the most prominent tinnitus frequency for each individual participant. The frequencies ranged from 170 to 16000 Hz. The sounds were played at an intensity based on the participant’s comfort level and adjusted for any hearing loss at different frequencies and intensities were limited to 80 dB SPL. Each of the tone frequencies was made to appear to arise from various 3D locations (programmed using a KEMAR head model) in order to avoid a bias of presenting a tone (paired with VNS) from a single spatial location. The frequency and intensity (dB SPL) of each tone were randomly selected each time a VNS pulse was delivered.

In the control (unpaired) group, VNS was not paired with tones (10 minutes of tones only, 5 minutes of silence and no VNS; 2 hours of VNS only; 5 minutes of silence and no VNS, and 10 minutes of tones only) during the 2.5-hour period (Fig. 5b).

### Delivery of Home-based Therapy

Both groups received therapy for 6 weeks (randomized portion of the study). The control group then crossed over to the paired VNS group settings after 6 weeks while the VNS group continued the original paired VNS therapy.

After the audiologist programmed the system settings, participants trained in proper use of the device to initiate daily stimulation at home. Although the stimulation was self-delivered by the participant at their home, a test therapy session was administered at the site under supervision of the investigator. VNS was administered to determine whether the participant could tolerate the standard settings and initiate therapy appropriately. If the participant was unable to tolerate the standard settings, output current was reduced from 0.8 mA in 0.1 mA steps until a tolerable level was reached. After a tolerable level of output current was obtained, the site operator verified that the participant could hear the tones and that the tones were coming from various locations as perceived through the headphones (site personnel would listen to the tones through the headphones and then have the participant listen and verify the tones could be heard).

Participants were contacted at least once during the first seven days of therapy so that the site could check that the participant was delivering therapy appropriately for the full 2.5-hour session. Participant compliance with performing at-home therapy was verified at sites by reviewing the data log files (log files had date/time/stimulation information), such that each participants daily use could be verified.

Participants were instructed to do therapy in a quiet room and to either read a magazine or book while sitting in a comfortable chair. They were also instructed not to sleep, watch TV (with audio), or have extended conversations during therapy. However, they were allowed to work on a muted computer. The intent was to allow the participant to hear the tones while still being able to perform some other tasks. Although participants were instructed to deliver therapy in their home at approximately the same time every day, they were allowed to deliver therapy at different times due to scheduling conflicts. If participants needed a bathroom break or if other social interruptions (i.e., telephone call) occurred, they were given a 15-minute break. Furthermore, if a participant missed more than an average of three therapy sessions a week, or more than 3 sessions in a row, that participant was eliminated from the primary analysis, but was included in any intent-to-treat analysis. Delivery of therapy was verified through review of the records on the participant’s computer at each study visit.

### Baseline Screening and Outcome Measures

**Figure 6:**
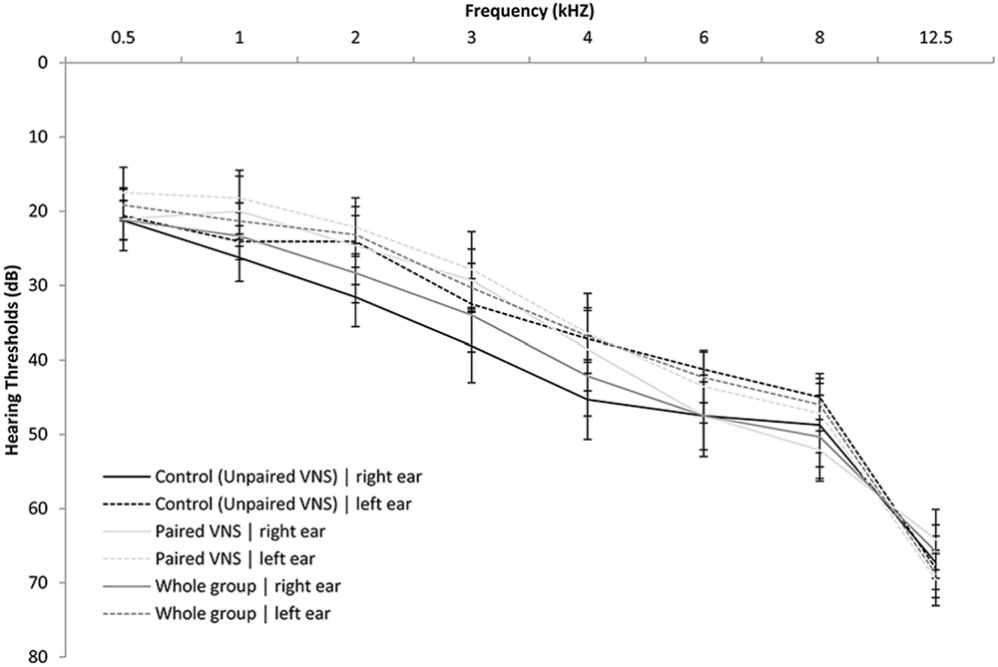
Pure Tone Audiometry for both the paired VNS and control group. Audiograms were obtained at baseline prior to implantation. Hearing thresholds were measured at 0.5 kHz, 1 kHz, 2 kHz, 3 kHz, 4 kHz, 6 kHz, 8 kHz, and 12.5 kHz.

Baseline screening included audiometry (through 12 kHz with pure tones for both air and bone conduction stimuli including monosyllabic word recognition testing) and tinnitus pitch matching. Hearing thresholds were measured at 0.5 kHz, 1 kHz, 2 kHz, 3 kHz, 4 kHz, 6 kHz, 8 kHz and 12.5 kHz (Fig. 6). Assessments included Tinnitus Handicap Inventory (THI), Tinnitus Handicap Questionnaire (THQ), Tinnitus Functional Index (TFI), loudness severity (participants were asked to rate the loudness of their tinnitus on a scale from 0–100; 0 meaning no tinnitus and 100 indicating loudest tinnitus that they can imagine), as well as psychoacoustic outcome measures including Minimum Masking Level (MML) and loudness matching^48,49^. Beck Depression Inventory (BDI)^50^, State-Trait Anxiety Inventory (STAI)^51^, and SF-12^52^ were also obtained. Since this was a pilot study, no primary outcome measure was designated. Participants had three baseline visits prior to starting therapy. The first visit was performed soon after consent was obtained to confirm eligibility. A second was performed at least two weeks later. Device implantation was then scheduled and participants were randomized to the paired VNS group or the control group (described below). A third baseline assessment was performed after surgery but prior to therapy initiation in order to determine whether surgery or anesthesia had any significant impact on assessments. All participants had their outcome measures administered in the clinic.

### Statistical Analysis

Baseline characteristics were displayed for each group by mean and standard deviation for continuous variables and numbers per category for categorical variables. Baseline characteristics were compared between groups using a t-test or a chi-squared test. Change from baseline was calculated for each group as percent or absolute change from baseline to 6 weeks. The within-group change in outcome (THI) from baseline to week 6 was analyzed with a one-sample t-test (H_0_: change = 0) and presented as a mean difference (with 95% confidence interval) for each group. Between group comparisons were made with two-sample t-test. For all comparisons, alpha was set at 0.05. No adjustments were made for multiplicity and each analysis was tested at the 0.05 level.

A repeated measures ANOVA was performed with condition (paired VNS vs. Control) as the between-subjects’ variable and the THI (at baseline, 6 weeks and 12 weeks) as the within-subject’s variable. A similar analysis was conducted for the THQ, TFI, loudness severity, MML and Loudness match. To test if the variance between the two groups (paired vs. control) was equal, we applied a Mauchly sphericity test. This test did not show statistically significant differences between the variances (Mauchly W = 0.84, χ^2^ = 4.79, *p* = 0.091).

A responder analysis was also performed to determine clinically meaningful effects. For the THI, a 20% reduction or an absolute 7-point reduction was considered clinically meaningful^29,30,52^. A 10-point reduction on the THQ and a 13- or 7-point decrease for the TFI were considered clinically meaningful^31,53^. To compare the responders and non-responders for both the paired VNS and control group, a χ^2^-coefficient was calculated. Statistical analysis was done using MATLAB (Mathworks, Inc.), SPSS and SAS v 9.4.

## Electronic supplementary material

Supplementary information
