# Supplementary material for: Vagus Nerve Stimulation Paired with Tones for the Treatment of Tinnitus: A Prospective Randomized Double-blind Controlled Pilot Study in Humans
Source: Sci Rep. 2017 Sep 20;7:11960. doi: 10.1038/s41598-017-12178-w (PMC5607328; doi:10.1038/s41598-017-12178-w)
Supplement: Supplementary file 1 — Supplementary information [file 41598_2017_12178_MOESM1_ESM.pdf]

Vagus Nerve Stimulation Paired with Tones for the Treatment of Tinnitus: A Prospective Randomized Double-blind Controlled Pilot Study in Humans

Richard Tyler[1*], Anthony Cacace[2], Christina Stocking[3], Brent Tarver[4], Navzer Engineer[4,] Jeffery Martin[5,] Aniruddha Deshpande[6], Nancy Stecker[3], Melissa Pereira[3], Michael Kilgard[7], Chester Buress[4], David Pierce[4], Robert Rennaker[7], Sven Vanneste[8]

Supplementary Methods

Audiometric Procedures

Pitch Matching: The most prominent pitch-match was obtained in the ear contralateral to the tinnitus ear in unilateral tinnitus participants, and contralateral to the worst tinnitus ear in bilateral tinnitus participants.

For the tinnitus pitch match, a paired-comparison procedure was used. First, 0.5 kHz and then a 4-kHz pulsed tone were presented and the participant was asked "Of the two tones presented to you, which has a pitch that is most like the most prominent pitch of your tinnitus?" If the participant said the higher tone, the next two tones compared would be 2 and 8 kHz. If the participant said the lower tone, the next two tones compared would be 0.25 and 1 kHz. Once the frequency region was established, half octave steps were used. When the participant reported the same tone as being similar twice, this value was recorded as the most prominent pitch. This sequence was repeated 3 times and the average recorded.

Loudness Severity: For the loudness severity, participants were asked to rate the loudness of their tinnitus on a scale from 0-100; 0 meaning no tinnitus and 100 indicating loudest tinnitus that they can imagine. This estimation was performed for both ears (or documented as only occurring in one ear).

Loudness Matching: For loudness matching, the estimation was performed for both ears (or documented as only occurring in one ear). Starting at 10 dB above the participants hearing threshold, they were asked to rate the loudness of their tinnitus compared to a 1000-Hz pulsed tone using an ascending method of limits. The tone was presented. Then, the participant was told "Tell me if your tinnitus is louder or softer than the loudness of my tone." If the participant said louder, the level was increased by 2 dB, if they said softer, the level was reduced by 2 dB. The test was stopped when the tinnitus loudness had been bracketed and the loudness recorded as the last test level (dB HL). This was repeated 5 times during each

measurement session and averaged to get a final value. The same 2-dB step size procedure was used to determine a 1000-Hz hearing threshold. This test was performed in the ear with tinnitus, or in the one with the loudest presentation if in both ears; if equal in both ears, the right ear was used.

Minimum Masking Level (MML): Measurements were made of the amount of noise required to mask a participant's tinnitus (Minimum Masking Level (MML)). With a pulsed (duration less than 5 seconds) broadband noise, the threshold and minimum masking level (MML) required to mask the tinnitus was measured with an ascending method of limits. First, the threshold for the noise was measured as described above with a 2 dB step size. For minimum masking level, the noise is presented at threshold and the participant asked "Tell me if you can hear your tinnitus above my noise." If the participant said yes, the noise was increased by 2 dB. The test was stopped when the tinnitus was masked. The last test level was the MML. This procedure was repeated 7 times and each participant's mean MML was used for comparisons. This was also repeated for the participant's other ear, and then documented in both ears for white noise.

Outcome measures

The THI [1] is a 25-item self-administered questionnaire that aims to quantify the impact of tinnitus on quality of life by measuring its effects on everyday function. Respondents are asked to answer the questions with 'Yes' (4 points) 'Sometimes' (2 points) or 'No' (0 points). A higher THI score (maximum 100) is indicative of a greater tinnitus handicap. The THI is a widely used, well validated and widely used outcome measure [2]. This study used THI as the primary outcome measure.

The THQ is a scale comprised of 27 items and is a well-established measure for the assessment of a broad spectrum of tinnitus-related psychological complaints [3]. Participants were asked to indicate agreement on a scale from 0 (if you strongly disagree) up to 100 (you strongly agree) [4-5].

The Tinnitus Functional Index (TFI) [6] is comprised of 25 items according to how they felt over the past week. Each item is scored on an 11-point scale with descriptors at either end of the scale. The sum of all scores is divided by 2.5 to give a global score out of 100. Higher scores reflect greater impact (worse) on daily functioning.

Adverse Event Reporting

All clinical signs and symptoms present at the time of enrollment and/or during the baseline period (prior to implantation) were documented. At the time of implantation and at each subsequent study visit, adverse events were recorded based on information volunteered by the participant and observations made by the health care staff. All events were recorded even if they were not considered to be related to study treatment, implantation, or the implantation system.

Sample size determination

This Pilot study was not powered to demonstrate statistically significant between-group results; it was based on typical sizes for IDE device pilot studies and was agreed upon with FDA. Typical study sizes for between-group differences of neurostimulation devices would be between 100 and 200 subjects. IDE pilot studies are typically between 10 and 50 subjects, and often do not have a separate control group (IDE pilot studies are often not randomized, parallel studies).

With respect to safety, a sample of 30 allowed adequate power to detect the incidence of rare safety and device events. A sample of 30 patients yields 95% probability that the study will reveal at least one occurrence of all events or complications that occur in patients at a rate of 9.5% or greater. In addition, implantation and follow-up of 30 patients for 6 weeks will yield 1,260 days of exposure. In this case, the threshold for detection decreases to a very unlikely event, one that occurs in only 0.2375% of days. In other words, if an event has a probability of 0.002375 or 1 event in 422 days of exposure we have a 95% probability of seeing this event in a sample of 30 patients with 1,260 days of exposure. As patients continue into the Long-Term portion of the study, the ability to detect rare events increases as the exposure increases.

Randomization Allocation Sequence

The CRO statistician created the randomization. Each site had one individual who was the "Programmer" that set the device to its correct settings. This was the only person at the site who was unblinded. The Programmer called a number at the Contract Research Organization (CRO) to receive the assignment as each subject to be randomized. Once proper identification information was given for both the Programmer and the Patient (Patient code and implant date), the Programmer was given the appropriate randomization

assignment. After the Programmer set the device to the appropriate group settings, they emailed a confirmation back to the CRO for final verification. The randomization code was not given to any other individual until the blind was broken at the end of the study.

Description of the similarity of interventions

The interventions were similar. All subjects had the same surgical procedure and device implanted. Both groups received vagus nerve stimulation and tones via headphones. The difference was in the timing and length of the stimulation and tones.

Recruitment dates

Recruitment started in February and March of 2014, with the first enrollment in April 2014. Recruitment continued through March 2015, with the last implant on March 25, 2015.

Supplementary Table 1. Enrollment Data at each clinical site

Study Site	Enrolled	D/C Prior to Implant	Implanted	Completed Acute Study
U. Iowa	11	3	8	8
UT-Dallas	25	16	9	9
U. Buffalo	6	0	6	6
Wayne State	20	13	7	7
**Totals**	**62**	**32**	**30**	**30**

NOTE: D/C = Discontinued Prior to Implant

Adverse Events

Of the 30 implanted subjects, 20 reported at least one adverse event, with similar numbers in each group (11 VNS subjects, 9 Control subjects). No new or unexpected events were reported. Events were typical of events reported for VNS in epilepsy and depression. Hoarseness after surgery was reported as described below. Other events included pain and soreness associated with surgery at the implant site, reaction to anesthesia (emesis/headache), fluid retention at implant site, muscle spasm, numbness, tingling, coughing, or sore throat during stimulation, and pain at high stimulation settings. All of these events were either mild or moderate. Two tables summarizing adverse events are included below in Supplementary Table 2

(stimulation) and Supplementary Table 3 (surgery). Four subjects reported hoarseness after surgery (two sites had one occurrence and one site had two occurrences). Two of the four patients had complete recovery within 12 weeks (no vocal cord paralysis was seen on laryngoscopy), while two of the four patients improved with compensation but still have some vocal breathiness. Since these last two subjects still had some vocal cord paralysis noted approximately 12 weeks after implant surgery, these were reported as serious adverse events. These two events, plus the surgery to replace the fractured lead, are the only three serious adverse events reported during the acute study. No new significant events related to the device were reported after the first 6-weeks. One subject (03-001) did have worsening of pre-existing depression; per physician feedback and MicroTransponder agreement the event is not thought to be due to VNS.

Supplementary Table 2. Adverse Events Possibly Related to Stimulation

Event	Total Reports	Number of Patients	Mild/Moderate
Coughing	Five (5)	Four (4)	4/1
Muscle Ache	Two (2)	Two (2)	2/0
Pain	Two (2)	Two (2)	0/2
Headache	One (1)	One (1)	1/0
Heartburn	One (1)	One (1)	0/1
Sore Throat	One (1)	One (1)	1/0
Nausea	One (1)	One (1)	1/0

Supplementary Table 3. Adverse Events Possibly Related to Surgery

Event	Total Reports	Number of Patients	Mild/Moderate
Incision Site Pain	Sixteen (16)	Ten (10)	13/3
Hoarseness[1]	Seven (7)	Seven (7)	5/2
Erythema	Three (3)	Three (3)	2/1
Constipation	Two (2)	Two (2)	1/1
Fluid at IPG site	Two (2)	One (1)	2/0
Muscle Twitch	Two (2)	Two (2)	2/0
Numbness	Two (2)	Two (2)	1/1
Neck Lump[2]	Two (2)	One (1)	2/0
Headache	One (1)	One (1)	0/1
Heartburn	One (1)	One (1)	1/0
Lightheadedness	One (1)	One (1)	1/0
Muscle Ache	One (1)	One (1)	1/0
Emesis	One (1)	One (1)	1/0

1 – Hoarseness due to either intubation (3) or surgery (4). Recovery was within one week for intubation, within 12 weeks for two due to surgery, and is ongoing in two cases (one year assessment has not yet occurred but is planned).
2 – Neck lump was reported both prior to surgery and after surgery in one subject, but was noted as possibly related to surgery.

Note: One lead fracture did occur and resulted in a 3-month delay in implants (reported to both the FDA and the study DSMB).

Supplementary Table 4. Responder rates for Paired VNS and Control group with previously reported cut-off values.

Outcome	Paired VNS (6 weeks)	Paired VNS (12 weeks)	Control (6 weeks)	Paired VNS after cross-over (12 weeks)
THI (>20 %) [a]	50%	56%	28%	43%
THI (>7 point) [b]	62.5%	62.5%	36%	50%
TFI (>7 point) [c]	37%	44%	28%	57%
TFI (>13 point) [d]	12.5%	12.5%	21.4%	50%

[a] Khedr, E. M., Rothwell, J. C., Ahmed, M. A. & El-Atar, A. Effect of daily repetitive transcranial magnetic stimulation for treatment of tinnitus: comparison of different stimulus frequencies. *J. Neurol. Neurosurg. Psychiatry* **79,** 212–215 (2008).

[b] Zeman, F. et al. Tinnitus handicap inventory for evaluating treatment effects: which changes are clinically relevant? Otolaryngol. -- Head Neck Surg. 145, 282–287 (2011).

[c] Folmer, R. L. et al. Repetitive Transcranial Magnetic Stimulation Treatment for Chronic Tinnitus: A Randomized Clinical Trial. JAMA Otolaryngol. Head Neck Surg. 141, 716–22 (2015).

[d] Meikle, M. B. et al. The tinnitus functional index: development of a new clinical measure for chronic, intrusive tinnitus. [Erratum appears in Ear Hear. 2012 May;33(3):443]. Ear Hear. 33, 153–176 (2012).

Supplementary Table 5. Long-term Responder rates for Paired VNS and Control group at 6 and 12 months (pooled data from participants receiving Paired VNS)

Outcome	Paired VNS (6 months) (n=subjects)	Paired VNS (1 year) (n=subjects)
THI (>20 %)	56% (n=16)	50% (n=16)
THI (>7 point)	62.5% (n=16)	50% (n=16)
TFI (>7 point)	52% (n=17)	50% (n=16)
TFI (>13 point)	32.5% (n=17)	37.5% (n=16)

Supplementary Table 6 Mean and Confidence interval data for anxiety and quality of life outcome measures at the end of the randomized portion of the study

Outcome	VNS (N=16)	Control (N=14)	Difference (Control - VNS)
Beck Depression Inventory	-0.07 (-3.33, 3.18)	0.14 (-1.56, 1.85)	0.21 (-3.28, 3.71)
SF12:Bodily Pain Transformed	9.38 (-0.20, 18.95)	3.57 (-6.00, 13.14)	-5.80 (-18.80, 7.19)
SF12:General Health Transformed	8.75 (-1.15, 18.65)	-1.79 (-7.95, 4.38)	-10.54 (-22.09, 1.02)
SF12:Mental Health Transformed	-3.57 (-13.55, 6.41)	-3.85 (-12.78, 5.08)	-0.27 (-13.07, 12.52)
SF12:Physical Functioning Transformed	1.56 (-9.81, 12.94)	1.79 (-5.07, 8.64)	0.22 (-12.97, 13.41)
SF12:Role Emotional Transformed	1.56 (-1.77, 4.89)	2.68 (-5.42, 10.77)	1.12 (-6.82, 9.06)
SF12:Role Physical Transformed	0.78 (-3.75, 5.31)	0.00 (-4.00, 4.00)	-0.78 (-6.64, 5.08)
SF12:Social Functioning Transformed	0.00 (-8.43, 8.43)	-1.79 (-8.64, 5.07)	-1.79 (-12.37, 8.80)
SF12:Vitality Transformed	0.00 (-9.81, 9.81)	-10.71 (-20.04, -1.39)	-10.71 (-23.59, 2.16)
STAI:S-Anxiety	-1.44 (-6.01, 3.14)	-0.07 (-5.06, 4.92)	1.37 (-5.09, 7.82)
STAI:T-Anxiety	-0.75 (-4.95, 3.45)	1.57 (-2.31, 5.45)	2.32 (-3.20, 7.84)

Supplementary References

[1] Newman, C. W., Jacobson, G. P., & Spitzer, J. B. (1996). Development of the Tinnitus Handicap Inventory. *Arch Otolaryngol Head Neck Surg, 122*(2), 143-148.

[2] Hall, D. A., Haider, H., Szczepek, A. J., Lau, P., Rabau, S., Jones-Diette, J., . . . Mazurek, B. (2016). Systematic review of outcome domains and instruments used in clinical trials of tinnitus treatments in adults. *Trials, 17*(1), 270. doi: 10.1186/s13063-016-1399-9

[3] Kuk, F. K., Tyler, R. S., Russell, D., & Jordan, H. (1990). The psychometric properties of a tinnitus handicap questionnaire. *Ear Hear, 11*(6), 434-445.

[4] Newman, C. W., Wharton, J. A., & Jacobson, G. P. (1995). Retest stability of the Tinnitus Handicap Questionnaire. Annals of Otology, Rhinology & Laryngology, 104, 718–723.

[5] Tyler, R. S. (1993). Tinnitus disability and handicap questionnaires. Seminars in Hearing, 14(4): 377-384.

[6] Meikle, M. B., Henry, J. A., Griest, S. E., Stewart, B. J., Abrams, H. B., McArdle, R., . . . Vernon, J. A. (2012). The tinnitus functional index: development of a new clinical measure for chronic, intrusive tinnitus. *Ear Hear, 33*(2), 153-176. doi: 10.1097/AUD.0b013e31822f67c0.
